# The effects of acute and chronic oral tea catechins and ornithine supplementation on exercise-induced ammonia accumulation and cycling performance in healthy young men: a randomized, double-blind, cross-over, placebo-controlled trial

**DOI:** 10.1007/s00421-025-05833-y

**Published:** 2025-06-12

**Authors:** Chihiro Nagayama, Yuka Hamada, Kayoko Kamemoto, Takahiro Hasumura, Yoshihiko Minegishi, Yuji Matsui, Noriyasu Ota, Masashi Miyashita

**Affiliations:** 1 Graduate School of Sport Sciences, Waseda University, Tokorozawa, Saitama Japan; 2 Institute for Sport Sciences, Waseda University, Tokorozawa, Saitama Japan; 3 Biological Science Research, Kao Corporation, Ichikai-Machi, Tochigi Japan; 4 Health & Wellness Products Research Laboratories, Kao Corporation, Sumida, Tokyo, Japan; 5 Faculty of Sport Sciences, Waseda University, Tokorozawa, Saitama Japan; 6 School of Sport, Exercise and Health Sciences, Loughborough University, Leicestershire, UK; 7 Department of Sports Science and Physical Education, The Chinese University of Hong Kong, Shatin, Hong Kong

**Keywords:** Ammonia metabolism, Ornithine, Exercise performance, Supplementation, Tea catechins

## Abstract

**Purpose:**

This study examined the effects of acute and chronic oral intake of tea catechins and ornithine supplementation on exercise-induced ammonia accumulation and cycling performance.

**Methods:**

Sixteen healthy young men participated in a randomized, double-blind, cross-over, placebo-controlled study. For the acute trials, the participants consumed either tea catechins and ornithine (CO) or placebo (P) and performed cycling exercises at an intensity corresponding to 75% of the maximum heart rate for 60 min, followed by a 15-min cycling performance test. The participants continued to consume each designated supplement for 13 days. For the chronic trials, the participants repeated the same protocol as the acute trials on day 14. After a washout period, the participants changed the supplement and repeated the same protocol as above.

**Results:**

Plasma catechins (acute ES = 3.61; chronic ES = 2.64, *p* < 0.001) and ornithine (acute ES = 4.28; chronic ES = 2.25, *p* < 0.001) concentrations were higher in both acute and chronic CO trials than those in P trials. No differences were found in plasma ammonia concentration measured during the whole experimental period and in mean power output during the performance test among trials. Subjective fatigue during 60-min cycling was lower in both acute and chronic CO trials than those in P trials (acute ES = 0.32, chronic ES = 0.60, *p* < 0.001).

**Conclusions:**

A single dose and 14-day oral intake of tea catechins and ornithine supplementation did not suppress exercise-induced ammonia accumulation or enhance cycling performance.

**Clinical trial:**

Clinical trial registration ID: UMIN000035267.

## Introduction

Ammonia is typically regarded as a toxic byproduct of amino acid metabolism and other nitrogenous compounds, and its efficient removal is crucial for maintaining physiological function (Wilkinson et al. 2010). It has been suggested that elevated ammonia concentrations during exhaustive exercise may impair the central nervous system and neuromuscular function, leading to fatigue and the inability to continue endurance exercise (Banister et al. 1983; Banister and Cameron 1990). Thus, any strategies to attenuate the exercise-induced ammonia accumulation may be of importance in preventing metabolic stress during strenuous exercise.

The nutritional supplementation strategy is one of the options for ameliorating the exercise-induced ammonia accumulation. For instance, tea catechins have been shown to stimulate the urea cycle, enhancing ammonia metabolism in mice (Chen et al. 2020). Moreover, tea catechin supplementation has been associated with improvements in aerobic capacity and exercise performance in humans (Ota et al. 2016; Yamagami et al. 2017, 2018), although its relationship with ammonia metabolism remains unclear. Ornithine is produced from arginine through arginase in the urea cycle and facilitates ammonia clearance, thus it is considered to be one of the important metabolites of the urea cycle to inhibit an increase in blood ammonia concentration induced by exercise (Takeda and Takemasa 2019). However, the impact of ornithine intake on ammonia metabolism and exercise performance remains contentious (Sugino et al. 2008; Demura et al. 2010, 2011). Nonetheless, research on the combined effects of tea catechins and ornithine remains limited. To our knowledge, only one study reported acute combined intake of tea catechins and ornithine for two consecutive days attenuated ammonia concentrations in response to cycling exercise (Hasumura et al. 2024), but did not measure circulating tea catechins and ornithine concentrations, limiting insights into bioavailability. Furthermore, it remains unknown whether acute vs chronic supplementation differently affects ammonia metabolism and exercise performance as the majority of previous single supplementation (i.e., either tea catechins or ornithine) studies did not distinguish acute or chronic influences (Sugino et al. 2008; Demura et al. 2010, 2011; Ota et al. 2016; Yamagami et al. 2017, 2018). Distinguishing these temporal effects is essential, as acute intake reflects immediate responses, whereas chronic intake may lead to cumulative physiological adaptations. Clarifying this distinction offers novel and practical insights into optimizing supplementation strategies.

Therefore, the present study aimed to assess the effects of both acute (i.e., a single dose) and chronic (i.e., 14 days) oral administration of tea catechins and ornithine supplementation on exercise-induced ammonia accumulation and cycling performance in healthy young men. We hypothesized that compared to the ingestion of dextrin as placebo, both acute and chronic oral supplementation of tea catechins and ornithine would attenuate ammonia concentrations and enhance cycling performance.

## Materials and methods

### Ethics approval

The present study was approved by the institutional ethics committee (approval number: 2018–201) and conducted in accordance with the Declaration of Helsinki. Participants of the present study were recruited between November 2018 and October 2019 through advertisements placed on the campus. Sixteen healthy men provided written informed consent to participate in the study. This study was registered in advance with the University Hospital Medical Information Network Center, a system for registering clinical trials (ID: UMIN000035267).

### Participants

**Fig. 1:**
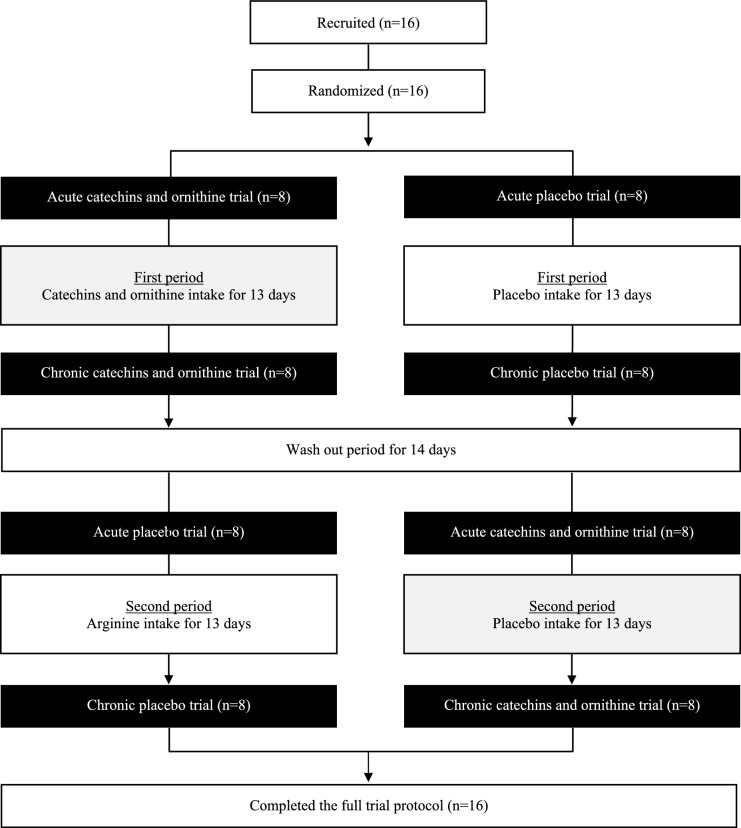
Consolidated standards of reporting trials (CONSORT) diagram showing the participant flow

A participant flow diagram is shown in Fig. 1. Individuals who are male, aged 20 to 39 years, engaged in exercise training at least three times a week were eligible (i.e., those who met all of the criteria) to take part in this study. Individuals were not eligible for participation if they were heart failure patients, had any chronic medical conditions, had a history of myocardial infarction, had a history of seizures based on nervous system diseases, had abnormalities of carbohydrate or lipid metabolism, were heavy alcohol drinker (20 g/day or more in terms of alcohol), were taking any supplementation or medication, were smoker, had difficulty exercising due to surgical injuries and medical disorders, had lost weight within the past 3 months and were planning to lose weight within the research period, and could not take the test drink. The physical characteristics of the participants (mean ± standard deviation) were as follows: age, 23 ± 3 years; height, 173.1 ± 4.6 cm; body mass, 64.6 ± 6.3 kg; body mass index, 21.6 ± 2.1 kg/m^2^; and maximum oxygen uptake, 52.0 ± 8.4 mL/kg/min. In this study, all data were collected in the laboratory of our academic institution from November 2018 to October 2019.

### Anthropometry

Body mass was measured to the nearest 0.1 kg using a digital scale (Inner Scan 50; Tanita Corporation, Tokyo, Japan) and height to the nearest 0.1 cm using a stadiometer (YS-OA; As One Corporation, Osaka, Japan). Body mass index was calculated as weight in kilograms divided by the square of height in meters.

### Preliminary tests

Participants participated in two preliminary exercise tests performed on a cycle ergometer (Monark 894E; Monark, Varberg, Sweden). A 16-min, four-stage, submaximal cycling test was conducted to determine the relationship between cycling workload and oxygen uptake. The initial cycling workload was set at 0.5 kg. The cadence of the cycle ergometer was set at 60 rpm throughout the test. The workload was increased by 1.0 kg every 4 min. After a 20-min rest (i.e., following completion of the submaximal cycling test), maximum oxygen uptake was measured directly with an incremental protocol until the participants reached volitional fatigue. The initial workload of the cycle ergometer was set between 2.0 and 3.5 kg depending on the fitness level of each participant obtained via interviews for this test. Thereafter, the workload was increased by 0.5 kg every 3 min (McArdle et al. 1973). Criteria used to confirm a maximum value included two of the following: (1) heart rate > 95% of age-predicted maximum heart rate (HRmax) and (2) ratings of perceived exertion (RPE) ≥ 19 using the Borg scale (Borg 1973). Oxygen uptake, carbon dioxide production and respiratory exchange ratio were measured breath-to-breath using a stationary gas analyzer (Quark CPFT; COSMED, Rome, Italy). Heart rate (HR) was monitored throughout these tests using short-range telemetry (Polar RCX3; Polar Electro, Kempele, Finland). Ratings of perceived exertion were assessed periodically during the tests using the Borg scale (Borg 1973). Data generated from these two tests were used to determine the cycling workload at 75% of each participant’s HRmax [75% of HRmax corresponded to 137 ± 7 beats per minute (bpm)], and this workload was used for the first main trial.

### Supplements

The composition of each supplement and its nutritional information are shown in Table 1. The supplements were powdered and prepacked in wrapping paper with no visible contents. For ingestion, one dose (12 g/pack) was dissolved in 200 mL of drinking water. Tea catechins are the major components of this extract and include epigallocatechin gallate (34.8%), epigallocatechin (34.4%), epicatechin gallate (9.8%), epicatechin (8.5%), gallocatechin (6.7%), catechin (2.9%), gallocatechin gallate (1.7%) and catechin gallate (1.1%). The dose of tea catechins (538.6 mg/200 mL) and ornithine (1592 mg/200mL) were chosen, since a previous study observed a suppressive effect of tea catechins and ornithine on exercise-induced increase in the blood ammonia concentration and no adverse effects in healthy young men when these individuals consumed the same dose for two consecutive days (Hasumura et al. 2024). The placebo supplement substituted tea catechins and ornithine with maltodextrin. Both supplements (i.e., test beverages) were indistinguishable in terms of volume, color, taste and flavor, ensuring a double-blinded approach.

**Table 1:** Components and nutrition facts of the supplements

	Catechins and ornithine	Placebo
(mg/200 mL)		
Tea catechins (total)	538.6	0
Epigallocatechin	187.7	0
Epigallocatechin gallate	170.5	0
Epicatechin	50.5	0
Epicatechin gallate	49.9	0
Gallocatechin	44.2	0
Catechin	16.7	0
Gallocatechin gallate	14.1	0
Catechin gallate	5.0	0
Ornithine	1592	0
Caffeine	8.5	10
(/200 mL)		
Energy (kJ)	179.9	190.4
Protein (g)	2.13	0
Fat (g)	0	0
Carbohydrate (g)	8.74	11.51

Caffeine was added to the placebo to compensate for the caffeine in the tea catechin formulation

### Study design and protocol

**Fig. 2:**
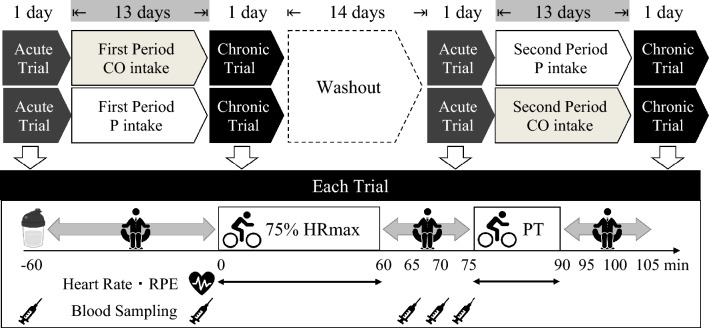
Schematic representation of the study protocol. Each participant underwent four, 1-day laboratory-based trials in a random order: (1) 1-day placebo (acute P trial), followed by (2) 14-day placebo (chronic P trial) and (3) 1-day tea catechins and ornithine (acute CO trial), followed by (4) 14-day tea catechins and ornithine (chronic CO trial) supplementation. Participants reported to the laboratory at 0850 h. After a 10-min seated rest, a fasting venous blood sample was collected (−60 min). For the acute trials, participants consumed 200 mL of water containing either tea catechins and ornithine (12 g) (CO) or placebo (12 g) (arginine replaced with maltodextrin). Further venous blood samples were collected immediately before cycling exercise (0 min), immediately post-cycling exercise (60 min), 5 min post-cycling exercise (65 min) and 10 min post-cycling exercise (70 min). Heart rate and ratings of perceived exertion (RPE) were measured continuously and periodically throughout both exercise sessions. From the day after each acute trial, the participants continued to consume each designated supplement twice a day for 13 days. For the chronic trials, the participants repeated the same protocol as the acute trials at day 14. After a 14-day washout period, the participants changed the supplement and repeated the same protocol as above. HRmax: maximum heart rate. PT: performance test

A randomized, double-blind, cross-over, placebo-controlled design was used in the present study. Each participant underwent four, 1-day laboratory-based trials in a random order: (1) 1-day placebo (acute P trial), followed by (2) 14-day placebo (chronic P trial) and (3) 1-day tea catechins and ornithine (acute CO trial), followed by (4) 14-day tea catechins and ornithine (chronic CO trial) supplementation. Trial order and randomization were selected from one of the two possible sequences using computer-generated random numbers in a counterbalanced manner to avoid order effects (performed the acute P trial, followed by the chronic P trial first or performed the acute CO trial, followed by the chronic CO trial first) by the lead investigator. To maintain the integrity of the study, a double-blind procedure was implemented. Neither the participants nor the researchers involved in data collection and analysis were aware of which treatment each participant received. In addition, the supplements were encoded with alphabet by the supplier of supplements (Kao Co., Ltd., Tokyo, Japan) in a manner that ensured both the participants and the researchers remained blinded throughout the study period. After data analysis was completed, the encoded data was opened at the laboratory of our academic institution. A schematic representation of the study protocol is shown in Fig. 2. Participants weighed and recorded all foods and drinks consumed the day before the first trial and replicated their dietary intake from the first trial in all subsequent trials to ensure that meals were standardized across trials. In addition, participants refrained from drinking alcohol for 2 days prior to each trial. Participants were also requested to remain inactive the day before each trial. Participants reported to the laboratory at 0850 h after a 10-h overnight fast (except water). After a 10-min seated rest, a fasting venous blood sample was collected by venipuncture at 0900 h (−60 min) to measure circulating concentrations of catechins, amino acids, ammonia and urea nitrogen. For the acute trials, participants consumed 200 mL of water containing either tea catechins and ornithine (12 g) (CO) or placebo (12 g). After a 60-min rest, the participants performed cycling exercise at an intensity corresponding to 75% of HRmax for 60 min, followed by a 15-min cycling performance test (Miyashita et al. 2019). There was a 15-min break between a 60-min fixed intensity cycling exercise and a 15-min cycling performance test. In the first main trial, the exercise workload for 60 min cycling was recorded and adjusted every 5 min to keep 75% of HRmax and this workload was used for remaining three trials. In the performance test, the participants were instructed to pedal a cycle ergometer (Monark 874E; Monark, Varberg, Sweden), exerting as much effort as possible at a self-selected pace. The work for each cycling performance test was calculated as the mean power output divided by each participant’s body mass using the Anaerobic Test Software (Monark ATS Software, Monark, Varberg, Sweden). Heart rate was monitored continuously using a short-range telemetry (Polar RS400; Polar Electro Oy, Finland) and RPE was evaluated every 5 min using Borg Scale (Borg 1973). Thereafter, participants were requested to sit in a chair (reading, writing or working on a computer) in the laboratory for 15 min. Further venous blood samples were collected immediately before cycling exercise (0 min), immediately post-cycling exercise (60 min), 5 min post-cycling exercise (65 min) and 10 min post-cycling exercise (70 min). Subjective fatigue was assessed using a visual analog scale for the four timepoints (at −60, 60, 75 and 90 min). From the day after each acute trial, the participants continued to consume each designated supplement twice a day (i.e., 12 g tea catechins and ornithine or placebo) for 13 days. The lead investigator asked each participant for their compliance in each main exercise day—all participants reported that they consumed all requested supplements during the supplementation period. For the chronic trials, the participants repeated the same protocol as the acute trials at day 14. After a 14-day washout period, the participants changed the supplement and repeated the same protocol as above. The primary outcome was time-averaged ammonia concentration determined at every blood sampling timepoint. The secondary outcomes were mean power output adjusted by body mass during the exercise performance test, time-averaged total catechins, amino acids and serum urea nitrogen concentration determined at every blood sampling timepoint, and HR and RPE during 60-min cycling exercise and exercise performance. No serious adverse events were observed during the study. In addition, none of participants dropped out from the study.

### Analytical methods

For serum urea nitrogen measurements, venous blood samples were collected into tubes containing clotting activators for serum isolation. Samples were allowed to clot for 30 min at room temperature and then centrifuged at 1861 × *g* for 10 min at 4 °C. For plasma ammonia measurements, venous blood samples were collected into tubes containing sodium fluoride–ethylenediaminetetraacetic acid (EDTA) and dipotassium salt–EDTA, and stored at 4 °C. Thereafter, serum and plasma samples were sent for further analysis (Kotobiken Medical Laboratories, Inc., Tokyo, Japan). For plasma selected amino acid fraction and catechins measurements (total catechin, epigallocatechin, epigallocatechin gallate, epicatechin, epicatechin gallate, gallocatechin, catechin, gallocatechin gallate and catechin gallate), venous blood samples were collected into tubes containing heparin–sodium EDTA and immediately centrifuged at 1861 × *g* for 10 min at 4 °C. Plasma was removed, divided into aliquots, and stored at −80 °C for later analysis. Enzymatic colorimetric assays were used to measure plasma ammonia (FUJI DRI–CHEM SLIDE NH3-PII; Fujifilm Co., Tokyo, Japan). Urease–glutamate dehydrogenase and ammonia removal methods were used to measure serum urea nitrogen (L type Wako-UN·V; FUJIFILM Wako Pure Chemical Co., Tokyo, Japan). Liquid chromatograph–mass spectrometry was used to analyze plasma concentrations of selected amino acid fraction. High-performance liquid chromatography was used to analyze plasma catechins concentrations as described previously (Umegaki et al. 2001). All analyses for each participant were completed within the same run for each measure. The intra-assay coefficients of variation were 3.6% for ammonia, 1.7% for urea nitrogen, 2.0% for amino acids, 3.0% for epigallocatechin gallate, 2.6% for epigallocatechin, 5.4%  for epicatechin gallate, 4.8% for epicatechin, 5.8% for gallocatechin, 5.2% for catechin, 4.1% for gallocatechin gallate and 4.9% for catechin gallate.

### Calculations and statistical analysis

We calculated the required sample size based on data from a previous study (Hasumura et al. 2024). The previous study reported the within-subject effect (effect size, Cohen’s *d* = 1.28 for the blood ammonia area under the curve) using a combination of tea catechins and ornithine vs placebo in response to a graded cycling exercise (Hasumura et al. 2024). For two trials with an alpha level set at 0.05 and a correlation of 0.867, an estimated total sample size of 11 would provide 80% power to detect between trial differences. We set the required participants to 16, since our study design was both acute and chronic supplementation interventions (i.e., for a total of 4 trials) and potential withdrawals were considered. Sixteen participants who completed the full trial protocol were included in each analysis. Data were analyzed with Predictive Analytics Software version 22.0 for Windows (SPSS Japan Inc., Tokyo, Japan). Normality of data was assessed using the Shapiro–Wilk test prior to statistical analysis, and all variables were confirmed to be normally distributed. The linear mixed model was used to examine between-trial differences over the 1-day or 2-week intervention for fasting serum or plasma concentrations, serum or plasma concentrations across four timepoints, visual analog scale, mean power output and HR values. Where significant trial or trial-by-time interaction effects were found, the data were subsequently analyzed using post-hoc analysis and were adjusted for multiple comparisons using the Bonferroni method. Statistical significance was accepted at the 5% level. The 95% confidence interval (CI) for the mean absolute pairwise differences between the trials was calculated using the t-distribution and degrees of freedom (*n* − 1). Absolute standardised effect sizes (ES) (Cohen’s d) are provided to supplement the findings. An ES of 0.2 was considered a small difference in all outcome measurements, 0.5 moderate and 0.8 large (Cohen 1988). Results are reported as the mean ± standard deviation.

## Results

### Total catechins and amino acids concentrations

The plasma total catechins and amino acids concentrations for each trial are shown in Table 2. Significant differences were found among trials in plasma total catechins, ornithine, citrulline, arginine, glutamic acid (all for *p* < 0.0001) and glutamine (*p* = 0.009) concentrations measured during the whole experimental period. No significant difference was observed among trials in plasma aspartic acid concentration measured during the whole experimental period (*p* = 0.080). Post-hoc analyses of the main effect of trial revealed that time-averaged plasma total catechins and ornithine concentrations were significantly higher in the acute CO trial than in the acute P trial (catechins: ES = 3.61; *p* < 0.001) (ornithine: ES = 4.28, *p* < 0.001), and significantly higher in the chronic CO trial than in the chronic P trial (catechins: ES = 2.64; *p* < 0.001) (ornithine: ES = 2.25; *p* < 0.001). Time-averaged plasma total catechins and ornithine concentrations were significantly higher in the chronic CO trial than in the acute CO trial (ES = 0.65, ES = 0.67, respectively; both for *p* < 0.01). Post-hoc analyses of the main effect of trial revealed that time-averaged plasma citrulline and glutamic acid concentrations were significantly higher in the acute CO trial than in the acute P trial (citrulline: ES = 0.90; *p* < 0.01) (glutamic acid: ES = 0.45; *p* < 0.05), and significantly higher in the chronic CO trial than in the chronic P trial (citrulline: ES = 0.54; *p* < 0.01) (glutamic acid: ES = 0.93; *p* < 0.001). Post-hoc analyses of the main effect of trial revealed that time-averaged plasma arginine and glutamine concentrations were significantly higher in the chronic CO trial than in the chronic P trial (arginine: ES = 0.75; *p* < 0.01) (glutamine: ES = 0.54; *p* < 0.05).

**Table 2:** Total catechins and amino acids concentrations measured at each timepoint in the catechins and ornithine trials and the placebo trials

							Whole experimental period				
			−60 min	0 min	60 min	70 min	Time averaged	Mean difference		95% CI of mean difference	Effect size (Cohen’s *d*)
Total catechins (ng/mL)	CO trial	Acute	0.4 ± 1.0	62.4 ± 27.5	92.0 ± 26.5		51.6 ± 13.7				
		Chronic	9.6 ± 13.9	69.1 ± 35.7	109.9 ± 31.9		62.9 ± 23.8	*a*	11.3	2.2 to 20.4	(0.65)**
	P trial	Acute	0.6 ± 1.5	0.4 ± 0.9	0.2 ± 0.4		0.4 ± 1.0	*b*	51.2	42.2 to 60.3	(3.61)***
		Chronic	0.0 ± 0.1	0.0 ± 0.1	0.0 ± 0.0		0.0 ± 0.1	*c*	62.9	53.8 to 71.9	(2.64)***
Ornithine (nM/mL)	CO trial	Acute	52.8 ± 13.3	134.0 ± 43.2	104.5 ± 22.9	95.0 ± 18.2	96.6 ± 17.4				
		Chronic	72.4 ± 37.2	150.7 ± 57.6	136.1 ± 53.7	122.3 ± 48.0	120.4 ± 42.1	*a*	23.8	12.5 to 35.1	(0.67)***
	P trial	Acute	50.6 ± 12.7	47.3 ± 11.6	45.0 ± 11.9	44.0 ± 11.0	47.1 ± 11.8	*b*	49.0	37.7 to 60.4	(4.28)***
		Chronic	44.8 ± 10.3	40.5 ± 9.2	39.5 ± 10.8	39.4 ± 10.5	41.3 ± 9.9	*c*	79.3	67.9 to 90.6	(2.25)***
Citrulline (nM/mL)	CO trial	Acute	30.9 ± 4.8	27.6 ± 3.9	35.3 ± 5.8	34.6 ± 5.5	32.1 ± 4.4				
		Chronic	31.5 ± 7.1	27.7 ± 4.1	34.8 ± 6.8	34.7 ± 7.9	32.2 ± 5.6	*a*	0.1	−2.0 to 2.1	(0.03)
	P trial	Acute	29.7 ± 4.6	26.4 ± 4.3	28.6 ± 7.3	28.4 ± 6.8	28.3 ± 5.3	*b*	3.7	1.7 to 5.8	(0.90)***
		Chronic	30.8 ± 6.3	26.4 ± 4.1	29.6 ± 7.2	30.9 ± 7.5	29.4 ± 5.5	*c*	2.7	0.7 to 4.7	(0.54)**
Arginine (nM/mL)	CO trial	Acute	99.8 ± 14.6	98.8 ± 9.8	92.7 ± 14.1	89.4 ± 14.2	95.2 ± 11.1				
		Chronic	101.0 ± 18.8	100.3 ± 15.3	97.5 ± 18.0	96.4 ± 19.9	98.8 ± 15.4	*a*	3.6	−1.7 to 9.0	(0.36)
	P trial	Acute	96.9 ± 12.1	92.1 ± 10.8	88.0 ± 13.0	88.8 ± 13.0	91.8 ± 11.4	*b*	3.6	−1.7 to 9.0	(0.26)
		Chronic	95.0 ± 12.8	88.3 ± 9.3	83.3 ± 15.0	89.0 ± 14.2	88.8 ± 10.7	*c*	9.9	4.6 to 15.3	(0.75)***
Glutamic acid (nM/mL)	CO trial	Acute	43.7 ± 21.5	34.7 ± 12.5	42.5 ± 20.5	42.7 ± 16.4	40.9 ± 15.4				
		Chronic	46.9 ± 15.1	38.2 ± 10.1	45.9 ± 17.9	43.5 ± 13.6	43.6 ± 11.1	*a*	2.7	−3.1 to 8.6	(0.15)
	P trial	Acute	32.9 ± 8.2	32.0 ± 13.9	36.8 ± 11.4	35.5 ± 15.8	34.4 ± 10.0	*b*	6.5	0.6 to 12.3	(0.45)*
		Chronic	36.4 ± 11.3	27.2 ± 10.1	37.2 ± 12.7	32.2 ± 15.1	33.3 ± 10.2	*c*	10.4	4.6 to 16.3	(0.93)***
Glutamine (nM/mL)	CO trial	Acute	553.9 ± 37.8	552.1 ± 43.1	539.5 ± 52.5	532.3 ± 51.9	544.5 ± 35.4				
		Chronic	571.5 ± 80.2	573.6 ± 63.6	565.8 ± 93.2	566.4 ± 71.2	569.3 ± 49.8	*a*	24.9	−0.5 to 50.2	(0.60)
	P trial	Acute	553.6 ± 38.8	544.8 ± 48.6	522.7 ± 54.7	537.5 ± 71.3	540.4 ± 44.9	*b*	4.2	−21.3 to 29.7	(0.10)
		Chronic	563.3 ± 78.9	539.6 ± 52.0	517.6 ± 81.1	555.2 ± 67.2	541.9 ± 57.8	*c*	26.0	0.5 to 51.4	(0.54)*
Aspartic acid (nM/mL)	CO trial	Acute	3.2 ± 1.3	2.8 ± 1.0	2.9 ± 1.0	3.0 ± 1.0	3.0 ± 0.8				
		Chronic	3.2 ± 1.6	3.1 ± 1.5	3.2 ± 1.6	3.0 ± 1.3	3.1 ± 1.4	*a*	0.1	−0.3 to 0.5	(0.10)
	P trial	Acute	2.8 ± 0.8	3.1 ± 1.4	2.8 ± 0.8	2.8 ± 1.0	2.9 ± 0.7	*b*	0.1	−0.3 to 0.5	(0.18)
		Chronic	2.9 ± 0.9	2.7 ± 0.9	2.8 ± 0.9	2.8 ± 0.9	2.8 ± 0.7	*c*	0.4	0.0 to 0.8	(0.29)

Values are mean ± standard deviation for *n* = 16. Means were compared using the linear mixed model. Post-hoc analysis was adjusted for multiple comparisons using the Bonferroni method. Post-hoc analysis of the main effect of trial; *a*: chronic CO vs acute CO, *b*: acute CO vs acute P, *c*: chronic CO vs chronic P

*p < 0.05, **p < 0.01, ***p < 0.001

*CI* confidence intervals, *CO* catechins and ornithine, *P* placebo

### Ammonia and urea nitrogen concentrations

The plasma ammonia and serum urea nitrogen concentrations for each trial are shown in Table 3. No significant difference was observed among trials in plasma ammonia concentration measured during the whole experimental period (*p* = 0.405). A significant difference was found among trials in serum urea nitrogen concentration measured during the whole experimental period (*p* < 0.001). Post-hoc analyses of the main effect of trial revealed that time-averaged serum urea nitrogen concentration was significantly higher in the chronic P trial than in the chronic CO trial (ES = 0.52; *p* < 0.05).

**Table 3:** Ammonia and urea nitrogen concentrations measured at each timepoint in the catechins and ornithine trials and the placebo trials

							Whole experimental period				
			−60 min	0 min	60 min	70 min	Time averaged	Mean difference		95% CI of mean difference	Effect size (Cohen’s *d*)
Ammonia (μg/dL)	CO trial	Acute	41.9 ± 20.5	33.6 ± 11.7	72.2 ± 21.9	40.6 ± 12.2	47.1 ± 9.4				
		Chronic	36.9 ± 6.5	31.7 ± 8.7	63.8 ± 21.1	40.6 ± 12.4	43.2 ± 8.3	*a*	−3.9	−10.5 to 2.7	(0.47)
	P trial	Acute	37.3 ± 11.1	31.5 ± 9.0	69.1 ± 21.7	39.3 ± 17.4	44.5 ± 11.1	*b*	2.7	−3.9 to 9.4	(0.24)
		Chronic	38.9 ± 12.5	29.5 ± 7.6	69.4 ± 26.5	37.2 ± 13.1	43.8 ± 12.1	*c*	−0.4	−7.1 to 6.2	(0.05)
Urea nitrogen (mg/dL)	CO trial	Acute	14.7 ± 3.3	14.4 ± 3.1	14.8 ± 2.9	14.9 ± 3.0	14.7 ± 3.1				
		Chronic	14.0 ± 3.7	13.6 ± 3.5	14.0 ± 3.7	14.0 ± 3.7	13.9 ± 3.6	*a*	−0.8	−1.7 to 0.1	(0.25)
	P trial	Acute	14.3 ± 3.0	14.0 ± 3.0	14.0 ± 2.8	14.4 ± 2.7	14.1 ± 2.9	*b*	0.6	−0.3 to 1.5	(0.22)
		Chronic	16.0 ± 4.0	15.7 ± 4.0	15.5 ± 3.9	15.4 ± 3.8	15.7 ± 3.9	*c*	−1.8	−2.7 to −0.9	(0.52)**

Values are mean ± standard deviation for *n* = 16. Means were compared using the linear mixed model. Post-hoc analysis was adjusted for multiple comparisons using the Bonferroni method. Post-hoc analysis of the main effect of trial; *a*: chronic CO vs acute CO, *b*: acute CO vs acute P, *c*: chronic CO vs chronic P

*p < 0.05, **p < 0.01, ***p < 0.001

*CI* confidence intervals, *CO* catechins and ornithine, *P* placebo

### The 60-min cycling exercise and cycling performance test

**Fig. 3:**
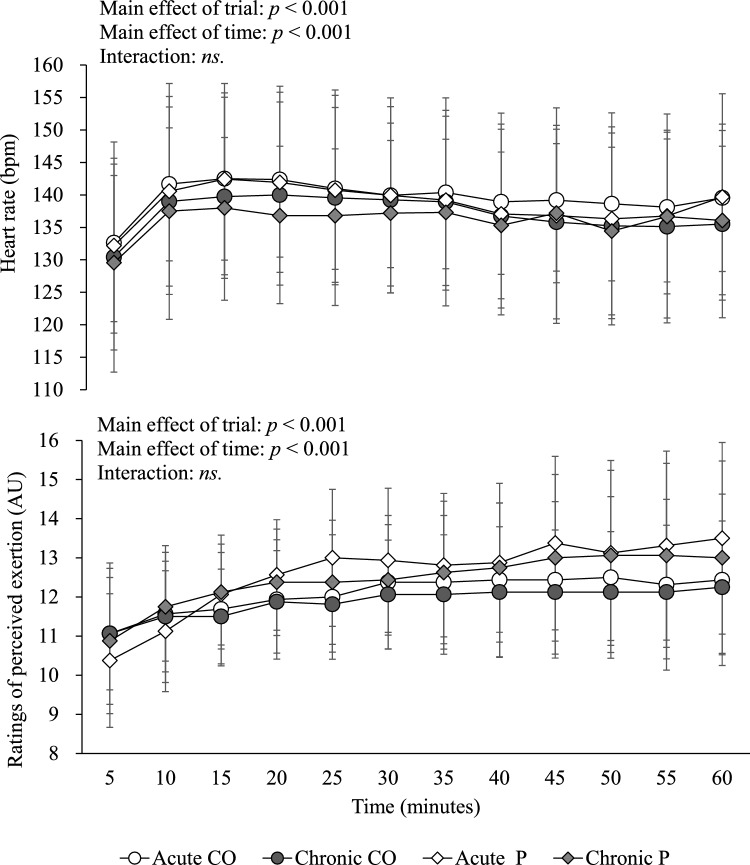
Heart rate and rating of perceived exertion during 60-min cycling exercise in the catechins and ornithine trials and the placebo trials. Data are means ± standard deviation for *n* = 16. Data were analyzed using the linear mixed model. Post-hoc analysis was adjusted for multiple comparisons using the Bonferroni method. Time-averaged heart rate was significantly higher in the acute catechins and ornithine (CO) trial than in both the chronic CO and placebo (P) trials. Time-averaged rating of perceived exertion was significantly lower in the acute CO trial than in both the acute and chronic P trials, and significantly lower in the chronic CO trial than in both the acute and chronic P trials

Heart rate and RPE during 60-min cycling exercise are shown in Fig. 3. Significant differences were found among trials in the HR and RPE during 60-min cycling exercise (both for *p* < 0.001). Post-hoc analyses of the main effect of trial revealed that time-averaged HR was significantly higher in the acute CO trial than in both chronic CO trial (Mean difference: 2.5 bpm, 95% CI 0.3–4.6 bpm, ES = 0.20, *p* = 0.014) and chronic P trial (mean difference: 3.5 bpm, 95% CI 1.4–5.6 bpm, ES = 0.42, p < 0.001). Post-hoc analyses of the main effect of trial revealed that time-averaged RPE was significantly lower in the acute CO trial than in both acute P trial (mean difference: −0.5, 95% CI −0.8 to −0.2, ES = 0.34, *p* < 0.001) and chronic P trial (mean difference: −0.4, 95% CI −0.7 to 0.0, ES = 0.30, *p* = 0.020), and significantly lower in the chronic CO trial than in both acute P trial (mean difference: −0.7, 95% CI −1.0 to −0.4, ES = 0.50, *p* < 0.001) and chronic P trial (mean difference: −0.6, 95% CI −0.9 to −0.2, ES = 0.57, *p* < 0.001).

**Fig. 4:**
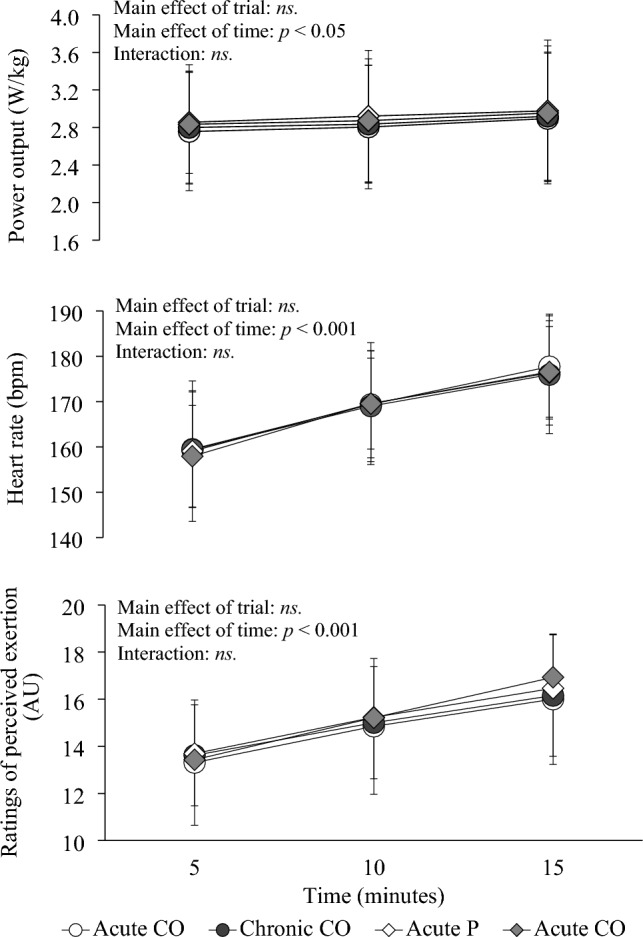
Mean power output, hear rate and rating of perceived exertion during a 15-min cycling performance test in the catechins and ornithine trials and the placebo trials. Data are means ± standard deviation for *n* = 16. Data were analyzed using the linear mixed model. Post-hoc analysis was adjusted for multiple comparisons using the Bonferroni method. No significant differences were observed among trials in power output, heart rate or rating perceived exertion during a 15-min cycling performance test. CO: catechins and ornithine. P: placebo

The mean power output, HR and RPE during a 15-min cycling performance test are shown in Fig. 4. No significant differences were observed among trials in power output, HR or RPE during a 15-min cycling performance test (*p* = 0.222, *p* = 0.941, *p* = 0.720, respectively).

## Discussion

The present study investigated the effects of acute and chronic oral administration of tea catechins and ornithine supplementation on exercise-induced ammonia accumulation and cycling performance in healthy young men. The main findings are as follows: (1) both acute (tea catechins 538.6 mg/200 mL and ornithine 1592 mg/200mL, 60 min prior to a 60-min cycling at an intensity corresponding to 75% of HRmax) and chronic (tea catechins 538.6 mg/200 mL and ornithine 1592 mg/200 mL for 14 days) oral administration of tea catechins and ornithine supplementation did not suppress exercise-induced ammonia accumulation or enhance cycling performance and (2) increased tea catechins and ornithine concentrations and lowered RPE were observed in both acute and chronic oral administration of tea catechins and ornithine supplementation.

Although a previous study found beneficial effects of combining tea catechins and ornithine on ammonia metabolism, the study lacked empirical data on the effect of this supplementation on their absorption in circulation (Hasumura et al. 2024). This study addressed this by demonstrating elevated plasma total catechins and ornithine concentrations in the CO trials compared to the P trials. In addition, plasma total catechins and ornithine concentrations were higher in the chronic CO trial than the acute CO trial, indicating that the dosage used can acutely elevate blood concentrations upon absorption and further increase them over a 14-day supplementation period. However, contrary to the hypothesis, the finding that oral administration of tea catechins and ornithine supplementation does not suppress ammonia accumulation induced by exercise is inconsistent with the findings of previous studies that investigated the effects of either single ornithine (Sugino et al. 2008; Demura et al. 2010) or a combination of tea catechins and ornithine administration (Hasumura et al. 2024). The differences in exercise load could contribute to this discrepancy between the studies. In the present study, the exercise regimen involved cycling at an intensity corresponding to 75% of HRmax for 60 min, which was relatively light compared to the exercise load used in the studies by Sugino and colleagues (Sugino et al. 2008) and Demura and colleagues (Demura et al. 2010). In the study by Sugino and colleagues, participants cycled at 80% of HRmax for 120 min, including 10-s maximum pedaling bursts (Sugino et al. 2008), while the study by Demura and colleagues employed an incremental exhaustive cycling exercise (Demura et al. 2010). In this study, the change in plasma ammonia concentration between baseline and peak values measured before and after exercise was approximately 30 μg/dL. This stands in contrast to previous research, where the magnitude of change ranged from approximately 90 to 150 μg/dL (Sugino et al. 2008; Demura et al. 2010). In addition, in a prior study by Hasumura and colleagues, participants engaged in cycling at 80% of HRmax for 40 min, exercise load similar to the present study (Hasumura et al. 2024). In this study, the observed change in ammonia concentration amounted to approximately 60 μg/dL, which is roughly twice that of our findings (Hasumura et al. 2024). Hence, the exercise loads employed in these previous studies (Sugino et al. 2008; Demura et al. 2010; Hasumura et al. 2024) seem to be more effective in triggering ammonia accumulation, and the considerable variability in blood ammonia could have influenced the dynamic change, since greater variability may allow for more substantial reductions. Moreover, the timing of blood collection during the recovery period after exercise could also be considered a contributing factor to the differences observed between the studies. It has been estimated that during exercise, only 10–25% of the ammonia produced in the muscle is released (Katz et al. 1986; Graham et al. 1990), suggesting that a significant proportion, possibly up to 90%, remains stored until the completion of exercise, after which it is gradually released and metabolized in the circulation during the recovery period (Graham et al. 1990). In this study, ammonia concentration was measured only up to 10 min after the cessation of fixed intensity cycling exercise, whereas in previous studies, blood ammonia responses during recovery were monitored for longer durations (ranged from 15 to 240 min) (Sugino et al. 2008; Demura et al. 2010; Hasumura et al. 2024).

The concentrations of intermediates in the urea cycle, including glutamine, glutamic acid, citrulline and arginine, were higher in the CO trials than the P trials in the present study. The intake of tea catechins or ornithine may potentially stimulate the upregulation of genes responsible for encoding enzymes in the urea cycle, resulting in elevated concentrations of amino acids, which are crucial intermediates in metabolic processes (Chen et al. 2020; Hasumura et al. 2024). The previous study demonstrated that the gene expressions of N-acetyl-glutamate synthetase, carbamoyl-phosphate synthetase 1, argininosuccinate lyase and arginase 1, which are key enzymes in the urea cycle, were increased in mice administered tea catechins for 4 weeks with regular treadmill running exercise (Chen et al. 2020). Furthermore, Hasumura and colleagues found that the activity of the urea cycle, which includes carbamoyl-phosphate synthetase 1, an essential enzyme for urea cycle regulation, increased when exposed to a combination of tea catechins and ornithine in hepatocyte-like cells derived from human induced pluripotent stem cells (Hasumura et al. 2024). However, this study observed a reduction in glutamic–oxaloacetic transaminase 1 (GOT1) expression in the tea catechins plus ornithine condition (Hasumura et al. 2024). Glutamic–oxaloacetic transaminase 1 is responsible for converting aspartate to oxaloacetate in the cytoplasm (Jiang et al. 2015). The authors speculated that the suppression of GOT1 by the combination of ornithine and tea catechins might lead to an increase in cytosolic aspartate concentration. This, along with the upregulation of other enzymes, could potentially enhance urea production under conditions of high ammonia and ornithine levels by facilitating the utilization of aspartate in the urea cycle (Hasumura et al. 2024). In the present study, the intake of tea catechins and ornithine did not alter plasma aspartic acid concentration (Table 2). Therefore, we speculate that the absence of changes in aspartic acid concentrations following tea catechins and ornithine intake might have contributed to the lack of ammonia detoxification and enhanced urea production observed in this study. However, the concentrations of plasma arginine, an intermediate metabolite before urea production in the urea cycle, were higher in the acute CO trial than in the chronic P trial and higher in the chronic CO trial than in both acute and chronic P trials in the present study. Therefore, the precise mechanism remains unknown.

The present study, to our knowledge, represents the first investigation into the combined effects of tea catechins and ornithine on exercise performance in humans. The findings, indicating that concurrent intake of tea catechins and ornithine did not improve cycling performance, are not consistent with those of previous studies that examined the effect of tea catechins or ornithine (Sugino et al. 2008; Demura et al. 2011; Yamagami et al. 2017, 2018). However, a direct comparison may not be feasible due to differences in study populations, performance testing protocols and outcomes. While no improvement in cycling performance was observed, our findings demonstrated that acute and chronic intake of tea catechins and ornithine reduced subjective fatigue during 60 min of cycling exercise. The ingestion of tea catechins and ornithine may have led to enhanced fatty acid β-oxidation during exercise, as well as increased oxygen intake (Murase et al. 2005, 2006; Ichinose et al. 2011; Takeda and Takemasa 2019; Willems et al. 2021), potentially resulting in reduced subjective fatigue during exercise. In addition, Sugino and colleagues reported that ornithine ingestion for 8 days (2,000 mg/day for 7 days and 6,000 mg/day for 1 day) attenuated the subjective feeling of fatigue in humans (Sugino et al. 2008). Conversely, a single oral administration of tea catechins did not result in a similar effect in mice (Haramizu et al. 2011), although the effect of a single oral administration of ornithine remains uncertain. Considering that plasma catechins and ornithine concentrations were also higher with chronic intake for 14 days in this study, repeated continuous intakes seem to be required to garner beneficial anti-fatigue effects of tea catechins and ornithine. Furthermore, it is important to consider that tea catechins are metabolized into various bioactive compounds after ingestion. In the context of chronic supplementation, the accumulation of catechin metabolites could have contributed to the observed reductions in subjective fatigue. Previous studies have reported that catechin metabolites accumulated following repeated green tea intake (Li et al. 2000) and continuous consumption of tea catechin was associated with enhanced recovery of oxygenated hemoglobin and myoglobin levels after cycling exercise in humans (Ota et al. 2016).

The present study has limitations to be considered. We did not conduct a familiarization session for the cycling performance test nor check the reliability/sensitivity of the cycling performance test protocol. Furthermore, the cycling performance test we used may not have elicited maximal effort from the participants effectively. Most participants were not highly trained cyclists, making it challenging to predetermine their maximum effort to cover the entire duration (i.e., 15 min) as they were asked to select a pedaling cadence freely at their own pace. These pre-conditional and training status may indeed influence the cycling performance test outcomes. Despite these limitations, the study has several strengths. We addressed the lack of empirical data on the effect of these supplements on their absorption in circulation. Our results indicate that the dosage used can acutely elevate blood concentrations upon absorption and further increase them over a 14-day supplementation period. In addition, the study evaluated both acute and chronic intakes of tea catechins and ornithine supplementation on exercise-induced ammonia accumulation and cycling performance. To date, this study is the first to compare these effects in a crossover study design. The unique nature of the study design enables us to distinguish between an acute and chronic intake concerning the magnitude of tea catechins and ornithine supplementation effects, if any, on these outcomes. Future studies are necessary to ascertain whether similar effects can be replicated across individuals of varying ages, genders, ethnicity and fitness levels, given that the present study exclusively involved young Asian men who were relatively physically fit.

## Conclusions

The present study showed that a single dose and 14-day oral intake of tea catechins and ornithine supplementation did not suppress exercise-induced ammonia accumulation or enhance cycling performance in healthy young men. However, subjective fatigue as reflected by the findings of lowered RPE during a 60-min fixed intensity cycling exercise was observed in both a single dose and 14-day oral administration of tea catechins and ornithine supplementation.

## Data Availability

The data are not publicly available but are available upon reasonable request to the corresponding author.

## Abbreviations

CI: Confidence interval
EDTA: Ethylenediaminetetraacetic acid
ES: Effect sizes
HR: Heart rate
HRmax: Maximum heart rate
CO: Catechins and ornithine
P: Placebo
RPE: Ratings of perceived exertion
